# Oxidative Stress Triggers Porcine Ovarian Granulosa Cell Apoptosis Through MAPK Signaling

**DOI:** 10.3390/antiox14080978

**Published:** 2025-08-09

**Authors:** Ting Zhao, Hui Jia, Xuerui Zhao, Xiaotong Gu, Chaoxiong Yong, Saihao Wang, Jiawei Zhou, Linrong Li, Mailin Gan, Lili Niu, Ye Zhao, Lei Chen, Xiaofeng Zhou, Linyuan Shen, Li Zhu, Yan Wang

**Affiliations:** 1 Farm Animal Genetic Resources Exploration and Innovation Key Laboratory of Sichuan Province, Sichuan Agricultural University, Chengdu 611130, China; zhaoting2@stu.sicau.edu.cn (T.Z.); jiahui@stu.sicau.edu.cn (H.J.); zhaoxuerui@stu.sicau.edu.cn (X.Z.); 2024202064@sicau.edu.cn (X.G.); 2024202016@sicau.edu.cn (C.Y.); 2023202048@sicau.edu.cn (S.W.); 2023002001@sicau.edu.cn (J.Z.); lilinrong@stu.sicau.edu.cn (L.L.); ganmailin@sicau.edu.cn (M.G.); niulili@sicau.edu.cn (L.N.); zhye@sicau.edu.cn (Y.Z.); chenlei815918@sicau.edu.cn (L.C.); zxf93715@163.com (X.Z.); shenlinyuan@sicau.edu.cn (L.S.); 2 State Key Laboratory of Swine and Poultry Breeding Industry, Sichuan Agricultural University, Chengdu 611130, China; 3 Key Laboratory of Livestock and Poultry Multi-Omics, Ministry of Agriculture and Rural Affairs, College of Animal and Technology, Sichuan Agricultural University, Chengdu 611130, China

**Keywords:** oxidative stress, granulosa cell, apoptosis, MAPK signaling pathway, ASK1, porcine follicle development

## Abstract

Follicle health determines the number and quality of sows’ ovulation, thereby influencing the litter size and the piglets’ viability. Granulosa cells (GCs) play a crucial role in follicular formation and development, and oxidative stress-induced GC death is a major cause of follicular dysplasia. Previous studies have confirmed that oxidative stress triggers apoptosis in granulosa cells. In this study, we explored how oxidative stress influences apoptosis in porcine ovarian granulosa cells. We find that porcine atretic follicles exhibit significant oxidative stress, accompanied by the activation of the mitogen-activated protein kinase (MAPK) signaling pathway, including the upregulation of key factors such as apoptosis signal-regulating kinase 1 (ASK1). Healthy follicles of 3–5 mm were randomly assigned to the control group, H_2_O_2_ treatment group, and selonsertib pretreatment group. The porcine ovarian GCs were placed in cell culture medium supplemented with H_2_O_2_ to assess ROS production, cell proliferation, apoptosis, the expression levels of oxidative stress-related genes, and expression levels of apoptosis-related proteins. In vitro experiments in mouse GCs further confirmed that H_2_O_2_-induced oxidative stress triggers the upregulation of the MAPK pathway and promotes granulosa cell apoptosis. The results showed that H_2_O_2_ treatment induced ROS production and apoptosis in porcine GCs and inhibited GC viability. Additionally, selonsertib pretreatment attenuated apoptosis in GCs by inhibiting H_2_O_2_-induced oxidative stress. In summary, our findings reveal that oxidative stress induced granulosa cell apoptosis via the MAPK signaling pathway, impairing proper follicular development in pigs.

## 1. Introduction

The reproductive potential of female animals is largely dependent on the critical step of follicle development and growth [1]. However, it is worth noting that the utilization rate of follicles in mammals is extremely low [2]. Only a small number of follicles can ultimately mature and complete the important process of ovulation, while the vast majority of follicles are unable to successfully develop beyond the follicular stage due to the widespread phenomenon of degeneration [3]. The direct consequence of this degeneration is the collapse of the follicular cavity and cell loss, which is academically referred to as follicular atresia [4]. Abnormal follicular atresia will significantly accelerate the consumption and exhaustion of follicular reserves, thereby causing reproductive function disorders and directly leading to a marked decline in female fertility and reproductive performance [5]. Follicular atresia is actually a natural physiological process that mammalian ovaries undergo, involving the spontaneous degeneration of ovarian follicles [6], which in turn impedes the normal growth and development of follicles [7]. Apoptosis of granulosa cells is a key factor in causing abnormal egg development and follicular atresia [8]. The loss of granulosa cells and the disruption of cell-to-cell communication may prevent the oocytes within pre-ovulatory follicles from obtaining adequate nutrition [9], greatly reducing their survival rate and ultimately leading to inevitable follicular atresia [10]. The apoptosis of granulosa cells is regulated by an array of factors [11], including hormones and cytokines, and is closely associated with changes in multiple signaling pathways [12].

Recently, an increasing body of research has highlighted the significant role of oxidative stress in follicular atresia and granulosa cell apoptosis [13]. Abnormal oxidative stress induces granulosa cell dysfunction and apoptosis [14]. Endogenous ROS are pivotal in regulating cellular processes such as growth, metabolism, differentiation, and apoptosis, as well as in modulating various signaling pathways [15]. However, an overabundance of ROS can lead to oxidative modifications of cellular macromolecules, thereby impairing protein function and potentially triggering cell death [16]. Furthermore, ROS function as signaling molecules that can stimulate both apoptosis and cellular differentiation [17]. Oxidative stress can trigger apoptosis in GCs, which are essential for the formation and development of ovarian follicles, as they surround the oocytes [18]. Despite the importance of these cells, the precise mechanisms by which oxidative stress induces apoptosis in GCs are not yet fully understood.

There is evidence suggesting that the accumulation of ROS, resulting from genotoxic chemicals, oxidants, nutrient deprivation, serum starvation, and other factors, can accelerate the apoptosis of GCs [19]. This process can lead to ovarian dysfunction, which includes conditions like premature ovarian aging (POA), premature ovarian failure (POF), and polycystic ovary syndrome (PCOS) [20]. It is now recognized that severe ovarian dysfunction can stem from oxidative damage to ovary tissues [21]. In contrast, specific inhibitors of oxidative stress, including estradiol and follicle-stimulating hormone (FSH), have demonstrated the ability to mitigate granulosa cell (GC) apoptosis during follicular atresia. [22]. Several studies have demonstrated that an increase in intracellular ROS levels, often induced by H_2_O_2_, can lead to DNA damage [23] and subsequent apoptosis [24]. However, the specific relationship between H_2_O_2_-mediated GC apoptosis and its broader implications for ovarian health requires further elucidation.

Here, this study aims to explore how oxidative stress affects the apoptosis of porcine ovarian granulosa cells. We found that there was significant oxidative stress in the abnormal follicles of pigs, accompanied by the activation of the MAPK signaling pathway. It includes the upregulation of key factors such as ASK1. To further verify this phenomenon, we randomly divided healthy follicles (with diameters of 3–5 mm) into a control group, an H_2_O_2_ treatment group, and a selonsertib pretreatment group. The ROS production, cell proliferation, and apoptosis of porcine ovarian granulosa cells, as well as the expression levels of oxidative stress-related genes and apoptosis-related proteins, were evaluated by adding H_2_O_2_ to the cell culture medium. Furthermore, our in vitro experiments in mouse granulosa cells further confirmed that H_2_O_2_-induced oxidative stress can activate the MAPK pathway and promote apoptosis in granulosa cells. Finally, we speculate that pretreatment with the ASK1 inhibitor selonsertib can effectively alleviate oxidative stress caused by H_2_O_2_, thereby reducing the apoptotic phenomenon of GCs.

## 2. Materials and Methods

### 2.1. Collection and In Vitro Culture of Follicles

Porcine ovaries from sexually mature sows were collected from a local slaughterhouse. The number of sows used was approximately 300. The sow ovaries were placed in 1×PBS containing streptomycin and penicillin, and follicles of 3–5 mm were selected for subsequent experimental treatment. Follicles were classified as healthy based on morphological criteria, including extensive vascularization of the follicular wall, translucent appearance with pink coloration, and clear follicular fluid, while atretic follicles exhibited pale coloration, reduced vascularity, and opaque follicular fluid [25,26]. Selected healthy follicles were individually placed in 48-well culture plates containing F12/DMEM medium supplemented with 15% fetal bovine serum and 2 mM L-glutamine. For experimental treatments, healthy follicles were randomly allocated into three groups: control group maintained in complete culture medium, oxidative stress group treated with 200 μM H_2_O_2_ for 12 h, and intervention group pretreated with 1 μM selonsertib for 1 h prior to H_2_O_2_ exposure. All cultures were maintained at 37 °C in a 5% CO_2_ cell culture chamber. The experiment was repeated three times.

### 2.2. Cell Culture

The mouse granulosa cell line was purchased from PriCells. Granulosa cells were cultured in DMEM/F12 medium containing 10% FBS, streptomycin (100 µg/mL), and penicillin (100 IU/mL). All cells were cultured at 37 °C in a 5% CO_2_ cell culture chamber. The H_2_O_2_ treatment group was treated with 200 µmol H_2_O_2_ for 12 h, and the pretreatment group was incubated with 1 μM selonsertib for 1 h prior to H_2_O_2_ exposure.

### 2.3. Antibodies

Antibodies against Phospho-ASK1 (Ser966) Rabbit pAb (310216), Cleaved-CASPASE 3 p17 Rabbit pAb (341034) were purchased from ZENBIO. Antibodies against Phospho-JNK1-T183/Y185 + JNK2-T183/Y185 + JNK3-T221/Y223 Rabbit mAb (AP1451), JNK2 Rabbit pAb (A1251), TRADD Rabbit pAb (A18626), MAP2K4 Rabbit pAb (A21597), and Phospho-MAP2K4-S257/T261 Rabbit pAb (AP0541) were purchased from ABclonal. Antibody against a-TUBULIN Mouse mAb (T9026) was purchased from Sigma.

### 2.4. Lipid Peroxidation Assay

The Reduced Glutathione (GSH) Colorimetric Assay Kit (E-BC-K030-M) was used to quantify peroxide levels through a colorimetric assay at 450 nm and to calculate the reduced GSH content [27]. Lipid peroxidation levels were assessed using the Lipid Peroxidation Assay Kit (Nanjing Jiancheng Bioengineering Institute, Nanjing, China, Catalog No. A003) according to the manufacturer’s protocol. Briefly, tissue homogenates or cell lysates were prepared and incubated with the provided reagents to generate a colorimetric product. The absorbance was measured at 586 nm using a spectrophotometer. All measurements were performed in triplicate to ensure data accuracy and reproducibility.

### 2.5. Malondialdehyde Colorimetric Assay

Malondialdehyde Colorimetric Assay Kit was used for measuring MDA accumulation by a colorimetric method (Nanjing Jiancheng Bioengineering Institute, Nanjing, China, Catalog No. A003-1).

### 2.6. Estradiol Test

A double-antibody sandwich enzyme-linked immunosorbent assay (ELISA) was performed to quantify estradiol levels in granulosa cells isolated from porcine healthy follicles and atretic follicles. Briefly, the samples to be tested were granulosa cells extracted from porker healthy follicles and abnormal follicles, and then anti-porker estradiol antibodies labeled with HRP were added (BY-JZF0264). Absorbance at 450 nm wavelength was determined by adding substrate A and B via an enzyme-labeled apparatus.

### 2.7. Serum Progesterone Test

The test samples were granulosa cells extracted from healthy and abnormal follicles, and then anti-porcine P4 antibodies labeled with HRP (BY-JZF1533) were added. After incubation and washing, substrates A and B were added. Absorbance was measured at 450 nm wavelength by an enzyme-labeled apparatus.

### 2.8. CCK-8 Assay

The CCK-8 assay was performed to assess cell viability at various time points during cell growth. Briefly, 10 μL of CCK-8 reagent (TargetMol, Shanghai, China) was added to each well containing 100 μL of culture medium in a 96-well plate. After incubating the plates at 37 °C for 2 h, absorbance was measured at 450 nm using a microplate reader (Thermo Scientific, Waltham, MA, USA).

### 2.9. DCFH-DA Assay

The fluorescent probe was first diluted at a ratio of 1:1000 in serum-free medium and subsequently added to the cell culture plates. Following a 30 min incubation at 37 °C, the medium was refreshed with complete medium. Finally, the cells were examined using a fluorescence microscope.

### 2.10. Mito Tracker Staining

To prepare the Mito Tracker solution, 94.06 µL of DMSO was added to a tube containing 50 µg of Mito Tracker powder to create a 1 mM storage solution [28]. This storage solution was then diluted to a working concentration of 500 nM using serum-free medium and applied to the cell culture plates. Following a 30 min incubation, the cells were washed twice with pre-warmed 1 × PBS (Servicebio, Wuhan, Hubei Province, China). The mitochondrial staining was subsequently visualized using a fluorescence microscope.

### 2.11. RNA Extraction and Quantitative RT-PCR

Total RNA was extracted using the RNAiso standard (Takara) according to the manufacturer’s protocol. cDNA was synthesized with Prime Script TM RT Reagent Kit with gDNA Eraser (Takara, Dalian, China) [29]. Gene expression levels were measured with iTaq^TM^ Universal SYBR^®^ Green Supermix (Bio-Rad, Hercules, CA, USA) on CFX96 (Bio-Rad). The relative expression levels of genes of interest were normalized to Actin expression. All experiments were repeated with at least three biological replicates, and the data are shown as mean ± standard error of the mean (SEM).

### 2.12. Total RNA Isolation and Quality Control

Total RNA extraction was performed using the RNAiso standard reagent (Takara, Dalian, China) following the manufacturer’s instructions. The quality of the extracted RNA was assessed via agarose gel electrophoresis, which was also used to purify RNA fragments of the desired size. In parallel, the RNA concentration was measured using a Nanodrop 2000 spectrophotometer (Thermo Fisher Science, Waltham, MA, USA).

### 2.13. RNA Annotation and Analyses for RNA-Seq Data

The sequencing platform used in this study was luminanovaseQ6000, and the sequencing service was provided by Novogene Co., Ltd. (Beijing, China). After obtaining the Raw Data, the Fastqc (v0.11.9) software is first used to detect the amount of sequencing data. If there are adapter sequences and N-bases, the Fastp (v0.23.2) software is then used for filtering and removal. The Clean Data obtained after filtering is used for subsequent analysis.

Before quantifying the Clean Data, the latest reference genome (Sscrofa11.1) file of pigs was obtained from the Ensmble database first. Then, the index was constructed using Hisat2 software, and Clean Data was aligned to the reference genome of pigs. After quantification using Kallisto (v0.44.0) software, the sequence reading results (Read Counts Matrix) obtained by quantifying all samples and the TPM standardized reading results (TPM Matrix) were integrated into corresponding matrices using a script written in R language. The subsequent analysis is based on the TPM Matrix.

### 2.14. Screening and Analysis of Differentially Expressed Genes

After obtaining the standardized transcriptome data, differentially expressed genes (DEGS) were screened for fold change > 1.5 or <0.67 with a *p* value < 0.05.

### 2.15. GO and KEGG Enrichment Analysis

We analyzed the differential genes of HF and AF and obtained the differential enrichment information in terms of biological processes, molecular functions, and cell composition through gene ontology (GO) analysis. Subsequently, these genes were also incorporated into the enrichment analysis framework of the Kyoto Encyclopedia of Genes and Genomes (KEGG) to reveal the signaling pathways related to the enrichment of differentially expressed genes. The specific analysis methods have been elaborated in detail in the previous research.

### 2.16. Western Blotting

Immunoblotting was performed as described in our previous study [30]. The ECL color developer was employed to visualize the protein bands, and the gray level of these bands was subsequently quantified using ImageJ 2.3.0/1.53q.

### 2.17. Statistical Analysis

Data analysis was conducted using GraphPad Prism 9.0 software, with results presented as the mean ± standard error of the mean (SEM). To assess significant differences between the two groups, Student’s *t*-test was employed, setting the significance level at *p* ≤ 0.05.

## 3. Results

### 3.1. Oxidative Stress as a Key Driver of Follicular Atresia

**Figure 1:**
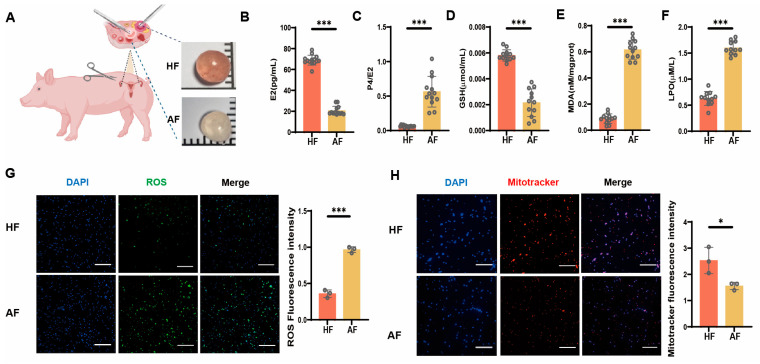
Oxidative stress occurs in porcine AF. (**A**) Comparison of morphology features between HF and AF. (**B**–**F**) The content of E2, P4/E2, GSH, MDA, and LPO in HF and AF. (**G**) ROS staining is used to detect reactive oxygen species in HF and AF. (**H**) Mito Tracker staining is used to detect mitochondrial activity in HF and AF. Scale bars, 100 µm. Data are presented as the mean ± SEM from a minimum of three independent biological replicates. Significance levels are indicated as follows: * *p* < 0.05 and *** *p* < 0.001.

The HF and AF of primary pigs were collected for observation, and a single follicle with a diameter of about 3–5 mm was isolated from the ovary. In a healthy follicle (HF), the follicular fluid is clear, and the follicular wall has sufficient blood vessels, which are pink. In an abnormal follicle (AF), the follicular walls are white and gray, the follicular fluid is turbid, and no obvious blood vessels are found (Figure 1A). To further investigate the difference between HF and AF, the study found that estradiol (E2) levels in atretic follicular fluid significantly decreased (Figure 1B). The ratio of progesterone (P4) to E2 significantly increased, indicating that growth of AF was restricted (Figure 1C). The GSH of AF decreased significantly (Figure 1D), and the activities of malondialdehyde (MDA) and lipid hydroperoxide (LPO) increased significantly (Figure 1E,F), suggesting that oxidative stress increased. Next, we performed ROS staining on HF and AF (Figure 1G). The results indicated that the levels of ROS in AF were markedly elevated compared to HF. Consistently, we used Mito Tracker dyes to stain active mitochondria; the fluorescence images showed that the positive signals of AF were significantly reduced (Figure 1H). These data indicate that compared with HF, oxidative stress occurs and increases in AF.

### 3.2. Transcriptomic Profiling of Porcine Healthy and Atretic Follicles

**Figure 2:**
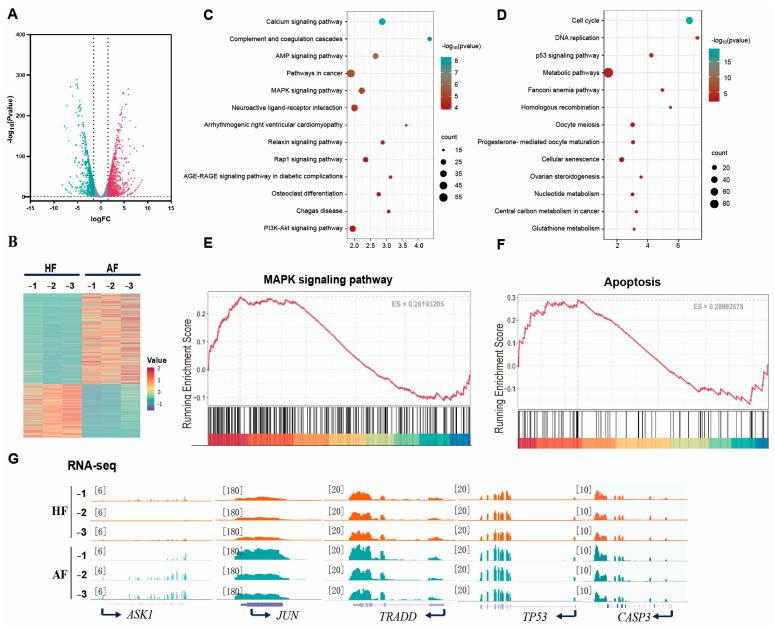
Bioinformatic analysis of RNA-seq data of porcine HF and AF. (**A**) Sequencing data variance volcano plots of HF and AF. (**B**) Sequencing data differential cluster heat map of HF and AF. (**C**) GO term enrichment analysis of functional annotation of different expression genes in HF and AF. (**D**) KEGG pathway analysis of different expression genes in HF and AF. (**E**,**F**) GSEA analyzed MAPK signaling pathway and apoptosis signaling pathway. (**G**) Genome browser tracks of RNA-seq ASK1, JUN, TRADD, TP53, and CASPASE3.

To characterize transcriptomic differences between healthy follicles (HF) and atretic follicles (AF), we performed RNA-seq analysis of GCs. Bioinformatic analysis of 15,167 detected mRNA transcripts identified 2477 differentially expressed genes (DEGs) (fold change ≥ 1.5 and *p*-value < 0.05), including 917 downregulated and 1560 upregulated transcripts (Figure 2A). All upregulated and downregulated mRNA transcripts were further evaluated by unsupervised hierarchical clustering (Figure 2B). The analysis showed that there was a good correlation between the groups, and there was a significant difference between HF and AF after analyzing the down- and upregulated genes in the GO and KEGG pathways in HF and AF. GO analysis identified the MAPK signaling pathway as the most significantly enriched biological process (Figure 2C). Consistent with these results, KEGG pathway analysis identified downregulation of oxidative stress-related pathways, particularly in metabolic and glutathione metabolism pathways (Figure 2D). These findings prompted focused investigation of two critical pathways: the MAPK signaling pathway (Figure 2E) and the apoptosis signaling pathway (Figure 2F), both known to mediate cellular responses to stress stimuli. Notably, our transcriptomic data revealed upregulation of the ASK1 signaling pathway in AF compared to HF (Figure 2G). This observation is particularly significant given the established role of MAPK pathways as central regulators of cellular responses to growth factors, nutrient availability, and various stress conditions [31]. The coordinated dysregulation of these pathways suggests their crucial involvement in oxidative stress-mediated granulosa cell dysfunction during follicular atresia.

### 3.3. MAPK Signaling Pathway Is Significantly Upregulated in AF

**Figure 3:**
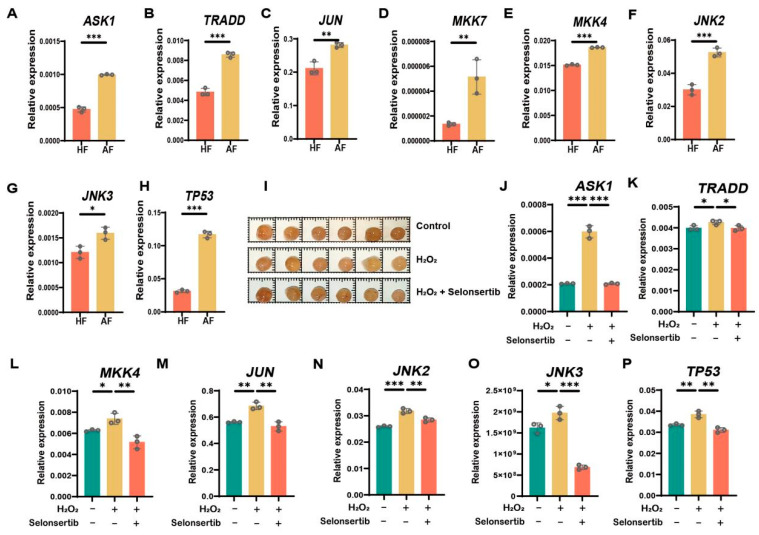
MAPK signaling pathway is significantly upregulated in porcine AF. (**A**–**H**) RT-qPCR analysis of mRNA expression levels of ASK1, TRADD, JUN, MKK7, MKK4, JNK2, JNK3, and TP53 in porcine HF and AF. (**I**) Healthy follicles of 3–5 mm were randomly assigned to the control group, H_2_O_2_ treatment group, and selonsertib pretreatment group. (**J–P**) The expression levels of ASK1, TRADD, JUN, MKK4, JNK2, JNK3, and TP53 in the control group, H_2_O_2_ treatment group, and selonsertib pretreatment group were detected by RT-qPCR. Data are presented as the mean ± SEM from a minimum of three independent biological replicates. Significance levels are indicated as follows: * *p* < 0.05, ** *p* < 0.01, and *** *p* < 0.001.

Previous studies have demonstrated that oxidative stress significantly reduces GC viability and induces apoptosis [32]. Quantitative PCR analysis revealed that the expression levels of ASK1, TRADD, JUN, MKK7, MKK4, JNK2, and JNK3 were significantly upregulated in GCs from AF compared to HF (Figure 3A–G), confirming activation of the ASK1 signaling pathway during follicular atresia. Notably, the apoptosis-related gene TP53 showed markedly higher expression in AF, consistent with increased apoptotic activity (Figure 3H). To establish a model of oxidative stress, we treated morphologically healthy follicles with 200 μM H_2_O_2_ for 12 h, a concentration that induced pathway activation without causing cell death. RT-PCR analysis confirmed that the ASK1 pathway was significantly upregulated within 12 h of H_2_O_2_ treatment, accompanied by increased TP53 expression, mirroring the gene expression pattern observed in natural atresia. The results demonstrate that oxidative stress induced GC cell apoptosis by upregulating the MAPK and ASK1 signaling pathways.

In addition, selonsertib is an ASK1 inhibitor that can block the ASK1 signal pathway. Therefore, selonsertib pretreatment alleviates the upregulation of ASK1 and apoptosis induced by GC by inhibiting H_2_O_2_-induced oxidative stress (Figure 3I–P). In summary, our study demonstrates that oxidative stress promotes follicular atresia by upregulating ASK1-dependent apoptosis in GCs, while selonsertib-mediated ASK1 inhibition provides a protective effect, suggesting potential therapeutic applications.

### 3.4. ASK1 Inhibition Rescued Oxidative Stress-Induced Apoptosis in Mouse GC

**Figure 4:**
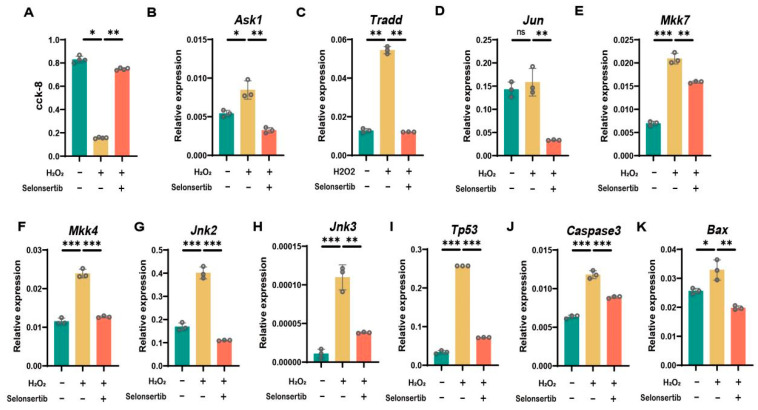
ASK1 inhibition protects mouse GCs from apoptotic cell death. (**A**) CCK-8 was used to detect the cell viability of the control group, H_2_O_2_ treatment group, and selonsertib pretreatment group. (**B**–**K**) The expression levels of ASK1, TRADD, JUN, MKK4, JNK2, JNK3, TP53, CASP3, and BAX in the control group, H_2_O_2_ treatment group, and selonsertib pretreatment group were detected by RT-qPCR. Data represented the mean ± SEM of at least three biological replicates. * *p* < 0.05; ** *p* < 0.01; *** *p* < 0.001; ns: not significant.

To further determine the effect of oxidative stress on follicular GC viability and apoptosis, we performed validation in mouse GCs. To avoid non-physiological cell death caused by excessive oxidation, mouse GCs were treated with 200 µmol H_2_O_2_ for 12 h. The CCK-8 assay verified that H_2_O_2_ treatment significantly reduced GC viability compared to controls (*p* < 0.01), while pretreatment with the ASK1 inhibitor selonsertib completely rescued this effect (Figure 4A).

Molecular analysis revealed that H_2_O_2_ treatment markedly upregulated key components of the ASK1 pathway, including ASK1, TRADD, JUN, MKK7, MKK4, JNK2, and JNK3 (Figure 4B–H). Importantly, selonsertib pretreatment effectively reversed these H_2_O_2_-induced changes, confirming ASK1 pathway inhibition. In addition, the expression levels of TP53, CASPASE-3, and BAX in GCs treated with H_2_O_2_ for 12 h were significantly higher than those in untreated GCs, and the pretreatment of selonsertib significantly reduced H_2_O_2_-induced apoptosis (Figure 4I–K), which was consistent with the vitality measurement.

**Figure 5:**
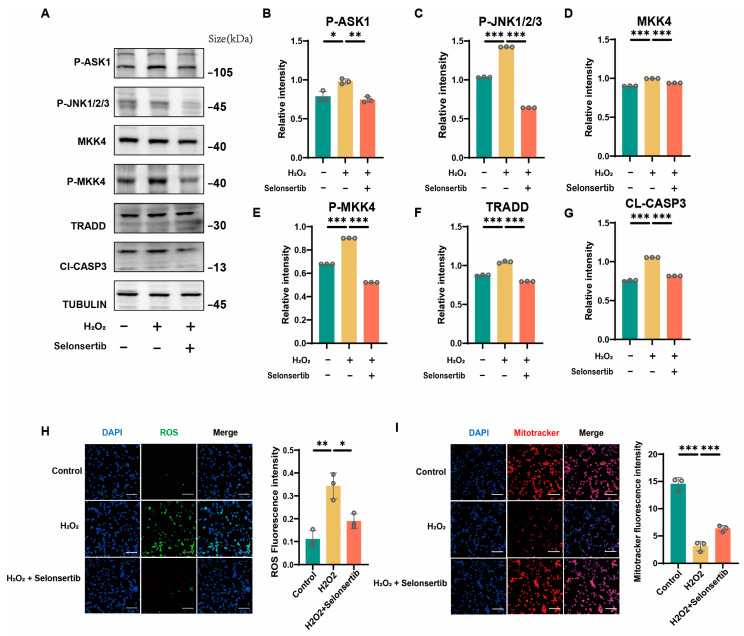
Inhibition of ASK1 rescued the apoptosis of GC. (**A**–**G**) Western blot was used to detect the expression levels of P-ASK1, TRADD, P-MKK4, JNK2, P-JNK1/2/3, and CL-CASP3 in the control group, H_2_O_2_ treatment group, and selonsertib pretreatment group. (**H**) ROS staining is used to detect reactive oxygen species in the control group, H_2_O_2_ treatment group, and selonsertib pretreatment group. (**I**) Mito Tracker staining is used to detect mitochondrial activity in the control group, H_2_O_2_ treatment group, and selonsertib pretreatment group. Scale bars in (**H**,**I**) are 100 µm. Data are presented as the mean ± SEM from a minimum of three independent biological replicates. Significance levels are indicated as follows: * *p* < 0.05, ** *p* < 0.01, and *** *p* < 0.001.

Moreover, Western blot analysis confirmed that protein-level expression patterns were consistent with transcriptional changes observed in our previous experiments (Figure 5A–G). Subsequent fluorescent staining assays provided direct evidence of oxidative stress induction in our model system. Specifically, H_2_O_2_-treated granulosa cells exhibited a dramatic increase in ROS accumulation, accompanied by an approximately 50% reduction in Mito Tracker fluorescence intensity (*p* < 0.01), indicating significant mitochondrial dysfunction (Figure 5H,I). Importantly, inhibition of ASK1 using selonsertib effectively mitigated these oxidative stress markers, reducing ROS levels and preserving mitochondrial membrane potential compared to H_2_O_2_ treatment alone. These findings provide compelling evidence that selonsertib protects granulosa cells by directly targeting the oxidative stress response at both the ROS production and mitochondrial integrity levels, ultimately preventing apoptosis through ASK1 pathway inhibition.

## 4. Discussion

Oxidative stress serves as a critical mediator of granulosa cell (GC) apoptosis, with well-documented pathological significance in follicular atresia [33]. ROS maintain essential physiological functions in ovarian follicular development at controlled concentrations, including regulation of meiotic resumption, ovulation, and corpus luteum dynamics [34,35]. However, an overproduction of ROS within follicles can lead to oxidative stress, which in turn can induce apoptosis in GC and result in follicular atresia [36]. Oxidative stress represents an imbalance between the generation of intracellular ROS and the cell’s antioxidant defenses [37]. In this study, we demonstrate that oxidative stress is involved in follicular atresia. In addition, H_2_O_2_ treatment can induce ROS production and apoptosis of porcine GC and inhibit the viability of granular cells. In addition, the pretreatment of selenium reduced the apoptosis of GC by inhibiting H_2_O_2_-induced oxidative stress. Our results establish a clear molecular link between oxidative damage and granulosa cell apoptosis while demonstrating the therapeutic potential of ASK1 inhibitors in ovarian dysfunction.

In cellular signaling, the MAPK pathway is a crucial mechanism through which cells respond to external stimuli, such as oxidative stress [38]. The MAPK family, which comprises extracellular signal-regulated kinases (ERK), c-Jun N-terminal kinases (JNK), and p38 MAPK isoforms [39], orchestrates fundamental cellular processes through precisely regulated phosphorylation cascades that ultimately determine cell fate, particularly apoptotic responses to oxidative insult [40]. ASK1, a critical member of the MAP3K family within the MAPK pathway, serves as a key regulator of apoptosis through its activation of downstream p38 and JNK signaling cascades [41]. Under conditions of oxidative stress, ASK1 activation occurs via multiple mechanisms, including redox-sensitive modifications and interactions with various adaptor proteins, in addition to phosphorylation by upstream kinases [42,43,44,45]. The resulting oxidative damage manifests through three principal mechanisms, including lipid peroxidation of cellular membranes, protein structure denaturation, and DNA strand breakage, collectively compromising cellular integrity and function [46,47]. H_2_O_2_ induces significant oxidative stress in GCs, characterized by increased ROS production, impaired mitochondrial function, and subsequent hyperactivation of the ASK1-MAPK signaling cascade, suggesting that excessive MAPK pathway activation may represent a key molecular mechanism underlying follicular atresia development.

GC is a cell type in the ovary that plays a crucial role in follicle development and maturation [48]. Under oxidative stress, GCs may undergo apoptosis, which is closely related to the activation of the MAPK pathway. Studies have shown that oxidative stress can activate the MAPK pathway, which in turn induces the upregulation of ASK1, leading to granulosa cell apoptosis. Our experimental results demonstrate that H_2_O_2_ treatment significantly induces ROS accumulation in both porcine follicles and mouse granulosa cells. Specifically, in the porcine follicle model, H_2_O_2_ exposure markedly elevated ROS levels in the follicular microenvironment while reducing antioxidant capacity in follicular fluid, accompanied by characteristic oxidative stress morphological changes in granulosa cells. In mouse GC cultures, H_2_O_2_ dose-dependently increased intracellular ROS production. Our data revealed conserved oxidative stress responses in mammalian GCs, with ROS accumulation showing a strong negative correlation with cell viability and parallel increases in apoptosis markers.

In summary, our study demonstrates that H_2_O_2_-induced oxidative stress triggers granulosa cell apoptosis through activation of the ASK1-MAPK pathway. We observed that oxidative stress conditions significantly increase reactive oxygen species production while simultaneously impairing mitochondrial function in cultured granulosa cells. Molecular analysis demonstrated marked upregulation of key apoptotic regulators, including TP53, CASPASE-3, and BAX, following oxidative challenge. Importantly, we identified that inhibition of ASK1 using selonsertib effectively protects granulosa cells by blocking this apoptotic cascade. The protective mechanism involves a substantial reduction in intracellular oxidative stress and preservation of mitochondrial membrane potential. These findings provide compelling evidence that targeted inhibition of the ASK1 pathway represents a viable therapeutic strategy for maintaining follicular health under oxidative stress conditions. These findings establish the ASK1 axis as a pivotal mediator of oxidative damage in follicular atresia and identify selonsertib as a potential therapeutic agent for ovarian dysfunction associated with oxidative stress.

## 5. Conclusions

**Figure 6:**
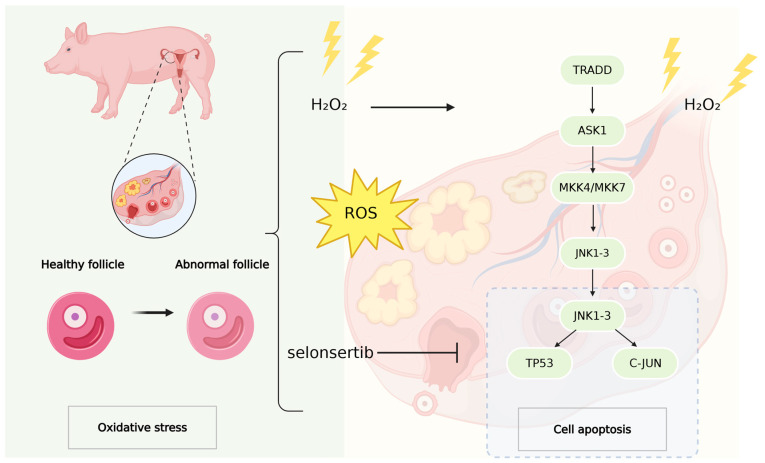
Oxidative stress-induced apoptosis is the main cause of granulosa cell death. This study aims to explore the effect of oxidative stress on the apoptosis of porcine ovarian granulosa cells. The ROS signal was elevated in abnormal follicles, and apoptosis increased. Oxidative stress-induced H_2_O_2_ treatment increased granulosa cell apoptosis. It was further proved that upregulation of ASK1 could induce apoptosis of GCs through the MAPK pathway. Furthermore, selonsertib pretreatment effectively protects GCs from oxidative stress by inhibiting the ASK1 pathway.

Our results highlight that oxidative stress induces the upregulation of apoptosis signal-regulating kinase 1 (ASK1) via the mitogen-activated protein kinase (MAPK) pathway, which subsequently may lead to granulosa cell apoptosis. This process is intricately linked with the interplay of multiple intracellular signaling pathways and transcription factors. By establishing the ASK1-JNK axis as a pivotal driver of oxidative stress-induced follicular atresia, our study not only advances the understanding of the molecular mechanisms underlying follicular dysfunction but also identifies selonsertib as a promising therapeutic candidate for addressing ovarian dysfunction mediated by redox imbalance(Figure 6).

## Data Availability

The datasets used or analyzed during the current study are available from the corresponding author upon reasonable request.
